# Our Principles

**Published:** 1913-06

**Authors:** 


### Department of Eugenics.

Conducted by Dr. Malone Duggan and Dr. Theo. Y. Hull.

### THE TEXAS STATE SOCIETY OF SOCIAL HYGIENE.

Headquarters: San Antonio, Texas.

Dr. Malone Duggan, President, San Antonio.
Dr. F. E. Daniel, 1st Vice President, Austin.
Prof. A. Caswell Ellis, 2d Vice President, Austin.
Mrs. Eli Hertzberg, 3d Vice President, San Antonio.
Dr. Theo. Y. Hull, Secretary, San Antonio.
Mr. W. L. Evans, Treasurer, San Antonio.

EXECUTIVE COMMITTEE.

Dr. Malone Duggan, Chm.
Dr. Theo. Y. Hull, Sec'y.
Dr. W. F. McCaleb.
Hon. C. H. Jenkins.
Rev. A. W. S. Garden.
Hon. J. C. Willacy.
Mr. W. L. Evans.
Dr. Frank D. Boyd.
Dr. J. R. Nichols.
Dr. J. M. McCutchan.
Dr. W. A. King.
Dr. J. W. Torbett.
Dr. F. C. Walsh.
Dr. Joe Reuss.
Dr. Frank Paschal.

### Our Principles.

Eugenics is a new religion. It is being accepted by thinking people with unprecedented enthusiasm. For that reason there is apt to be some misunderstanding as to what it truly teaches and what it does not teach. There is the scientific side and the practical side. This department, in the main, will confine its work to the practical side—the application of the principles of Eugenics in the intrinsic development of mankind.

Our Motto: The Child Well Born.

Our Creed: We believe in the exaltation of womanhood, and the protection of motherhood by fatherhood. We believe in the virtue and virility of woman as the type of the race. We believe in the continence of man as necessary to the highest aim of reproduction, and we believe in the responsibility of parenthood.

Our Slogan: (1) The deliberate and intelligent control of the offspring. (2) Marriage certificate showing freedom from venereal infection. (3) The sterilization of the feeble-minded, the degenerate and criminal. (4) The prevention of venereal diseases.

Our Program: Through every means possible to give instruction in a pure sex hygiene in the home, in the school and in the State, to the end that every man and woman may better know the nature and purpose of the sex instinct, thereby possessing them with the hidden secret of how to build happy homes and increase their efficiency.

Our Apology: We have none. Our high aim speaks for itself. There are those who may think such views too radical. The world was not made in a day, much less are old prejudices uprooted in a generation. New facts must wait for adjustment. But just so sure as the stone wears away under the constant dripping of water so will prejudice dissolve under the illumination of truth. Already the dawn of the new era has risen over the eastern horizon, and ere its setting rays glint the western seas, the mission of Eugenics will have been fulfilled.

